# 3,3′-Bis(4-chloro­phen­yl)-2,2′-(*m*-phenyl­enedi­oxy)diquinazolin-4(3*H*)-one

**DOI:** 10.1107/S1600536808040567

**Published:** 2008-12-10

**Authors:** Hai-Zhou Yang, Hai-Tao Gao, Xu-Hong Yang

**Affiliations:** a Department of Physical Education, Xianning College, Xianning 437100, Hubei, People’s Republic of China; b Department of Medicinal Chemistry, Yunyang Medical College, Shiyan 442000, Hubei, People’s Republic of China; c Department of Chemistry and Life Science, Xianning College, Xianning 4371000, Hubei, People’s Republic of China

## Abstract

In the title compound, C_34_H_20_Cl_2_N_4_O_4_, the two quinazoline heterocyclic systems and the adjacent chloro­benzene rings are not coplanar, but oriented at dihedral angles of 66.66 (13) and 52.48 (12)°, respectively. The quinazoline ring systems are nearly planar, with dihedral angles between the planes of the two rings of 5.43 (16) and 3.40 (14)°, and are oriented  at dihedral angles of 79.73 (13) and 83.52 (13)° with respect to the adjacent benzene ring between them. Inter­molecular C—H⋯O hydrogen bonds contribute to the stability of the structure. In addition, weak π–π stacking inter­actions [centroid-to-centroid distances = 3.872 (1) and 3.876 (1) Å] are observed in the crystal structure.

## Related literature

Many derivatives of quinazoline-4(3*H*)-one have been prepared, and their biological properties, such as anti­microbial, anti­diabetic, anti­convulsant, anti­bacterial and anti­fungal activities, and their action as protein tyrosine kinase inhibitors, EGFR inhibitors and PDGFR phospho­rylation inhibitors, have been studied by: Pandeya *et al.* (1999 ▶); Shiba *et al.* (1997 ▶); Malamas & Millen (1991 ▶); Mannschreck *et al.* (1984 ▶); Kung *et al.* (1999 ▶); Bartroli *et al.* (1998 ▶); Palmer *et al.* (1997 ▶); Tsou *et al.* (2001 ▶); Matsuno *et al.* (2002 ▶). For the synthesis, see: Yang *et al.* (2008 ▶). For related structures, see: Hu *et al.* (2006 ▶); Qu *et al.* (2008 ▶); Zeng *et al.* (2008 ▶); Sun *et al.* (2008 ▶).
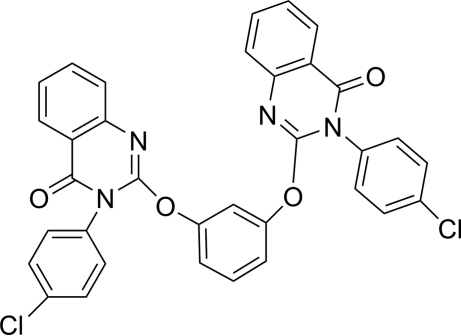


## Experimental

### 

#### Crystal data

C_34_H_20_Cl_2_N_4_O_4_
*M* *_r_* = 619.44
Monoclinic, 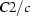

*a* = 28.043 (2) Å
*b* = 11.3563 (8) Å
*c* = 21.5497 (16) Å
β = 122.7440 (10)°
*V* = 5772.2 (7) Å^3^
*Z* = 8
Mo *K*α radiation
μ = 0.27 mm^−1^
*T* = 296 (2) K
0.23 × 0.10 × 0.10 mm

#### Data collection

Bruker SMART APEX CCD area-detector diffractometer
Absorption correction: none
22711 measured reflections
6251 independent reflections
3432 reflections with *I* > 2σ(*I*)
*R*_int_ = 0.069

#### Refinement

*R*[*F* ^2^ > 2σ(*F* ^2^)] = 0.066
*wR*(*F* ^2^) = 0.160
*S* = 1.01
6251 reflections
397 parameters
H-atom parameters constrained
Δρ_max_ = 0.23 e Å^−3^
Δρ_min_ = −0.28 e Å^−3^

### 

Data collection: *SMART* (Bruker, 2000 ▶); cell refinement: *SAINT* (Bruker, 2000 ▶); data reduction: *SAINT*; program(s) used to solve structure: *SHELXS97* (Sheldrick, 2008 ▶); program(s) used to refine structure: *SHELXL97* (Sheldrick, 2008 ▶); molecular graphics: *SHELXTL* (Sheldrick, 2008 ▶); software used to prepare material for publication: *SHELXTL*.

## Supplementary Material

Crystal structure: contains datablocks global, I. DOI: 10.1107/S1600536808040567/at2685sup1.cif

Structure factors: contains datablocks I. DOI: 10.1107/S1600536808040567/at2685Isup2.hkl

Additional supplementary materials:  crystallographic information; 3D view; checkCIF report

## Figures and Tables

**Table 1:** Hydrogen-bond geometry (Å, °)

*D*—H⋯*A*	*D*—H	H⋯*A*	*D*⋯*A*	*D*—H⋯*A*
C20—H20⋯O2^i^	0.93	2.34	3.234 (3)	162

Symmetry code: (i) 
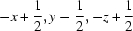
.

**Fig. 1.**
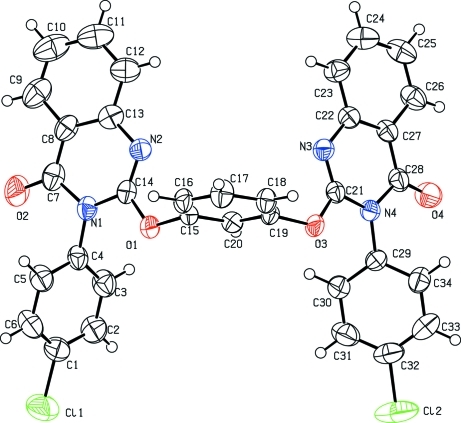
View of the molecular structure of (I), showing the atom labelling scheme and with displacement ellipsoids drawn at the 50% probability level.

**Fig. 2.**
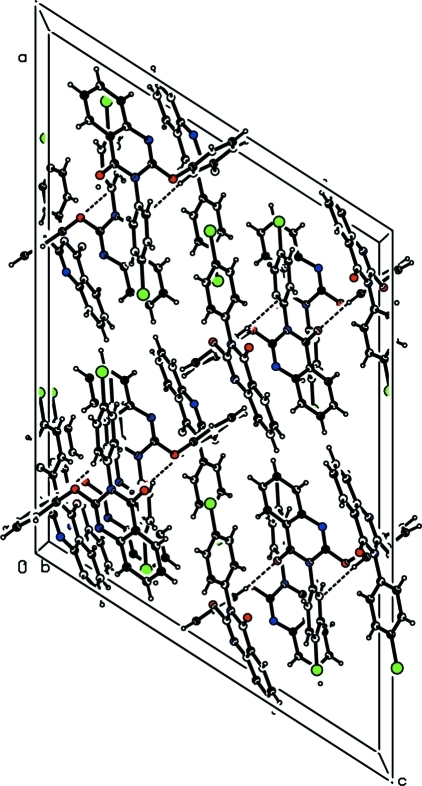
A partial view of the crystal packing of (I), showing the formation of C—H···O hydrogen-bonds, as dashed lines.

## supplementary crystallographic information

### Comment

Quinazoline derivatives have broad biological properties. Some of these activities include antimicrobial (Pandeya *et al.*, 1999; Shiba *et al.*, 1997), antidiabetic (Malamas & Millen, 1991), anticonvulsant (Mannschreck *et al.*, 1984), antibacterial (Kung *et al.*, 1999), antifungal (Bartroli *et al.*, 1998), protein tyrosine kinase inhibitors (Palmer *et al.*, 1997), EGFR inhibitors (Tsou *et al.*, 2001) and PDGFR phosphorylation inhibitors (Matsuno *et al.*, 2002). We have recently focused on the synthesis of heterocyclic compounds using an aza-Wittig reaction. We present here the synthesis and the crystal structure of the title compound, (I) (Fig. 1), which can be used as a precursor for obtaining bioactive molecules.

In the crystal structure of (I), the quinazoline heterocycle N1—C7/C8–C13/N2—C14 and N3—C22/C23–C28/N4—C21 and the adjacent chlorobenzene ring C1–C6 and C29–C34 are not co-planar, but oriented at the dihedral angles of 66.66 (13) and 52.48 (12)°, respectively. The nearly planar quinazoline ring system N1—C7/C8–C13/N2—C14 and N3—C22/C23–C28/N4—C21 are oriented with respect to the adjacent ring C15–C20 at the dihedral angles of 79.73 (13) and 83.52 (13)°, respectively.

Significant intramolecular C—H···O hydrogen bonds contribute to the stability of the molecular configuration (Fig. 2 and Table 1). The crystal structure (Fig. 2) is also stabilized by weak π–π (Table 1) stacking interactions with centroid–centroid separations of 3.872 (1) and 3.876 (1) Å for Cg2···Cg2^i^ and Cg2···Cg6^i^, respectively, where Cg2 and Cg6 are the centroids of rings N3/C21—N4/C28—C27/C22 and C22–C27,  respectively [symmetry code: (i) 1 - x, -y, 1 - z].

### Experimental

To a solution of iminophosphorane (1.40 g, 3.0 mmol) in anhydrous THF (10 ml) was added 4-chlorophenyl isocyanate (3 mmol) under nitrogen at room temperature. After reaction, the mixture was allowed to stand for 10 h at 273–278 K, the solvent was removed under reduced pressure and diethyl ether/petroleum ether (1:2 *v*/*v*, 20 ml) was added to precipitate triphenylphosphine oxide. After filtration, the solvent was removed to give 1-(4-chlorophenyl)-3-(2-ethoxycarbonylphenyl) carbodiimide, which was used directly without further purification. To a solution of 1-(4-chlorophenyl)-3-(2-ethoxycarbonylphenyl) carbodiimide in THF (15 ml) was added *m*-dihydroxybenzene (0.18 g, 3 mmol). After the reaction mixture was allowed to stand for 0.5 h, the solvent was removed and anhydrous ethanol (10 ml) with several drops of EtONa in EtOH was added. The mixture was stirred for 2 h at room temperature. The solution was concentrated under reduced pressure and the residue was recrystallized from ethanol to give the title compound, (I). The product was recrystallized from methanol–dichloromethane (1:1 *v*/*v*, 20 ml) at room temperature to give crystals suitable for X-ray diffraction [m.p. 444 K, yield 45%].

### Refinement

All H atoms were located in difference maps and treated as riding atoms with C—H = 0.93 Å, *U*_iso_ = 1.2*U*_eq_(C) for C*sp*^2^.

### Crystal data

**Table:**

C_34_H_20_Cl_2_N_4_O_4_	*F*(000) = 2544
*M**_r_* = 619.44	*D*_x_ = 1.426 Mg m^−^^3^
Monoclinic, *C*2/*c*	Mo *K*α radiation, λ = 0.71073 Å
Hall symbol: -C 2yc	Cell parameters from 1972 reflections
*a* = 28.043 (2) Å	θ = 2.3–19.8°
*b* = 11.3563 (8) Å	µ = 0.27 mm^−^^1^
*c* = 21.5497 (16) Å	*T* = 296 K
β = 122.744 (1)°	Block, colourless
*V* = 5772.2 (7) Å^3^	0.23 × 0.10 × 0.10 mm
*Z* = 8	

### Data collection

**Table:**

Bruker SMART APEX CCD area-detector diffractometer	3432 reflections with *I* > 2σ(*I*)
Radiation source: fine-focus sealed tube	*R*_int_ = 0.069
graphite	θ_max_ = 27.0°, θ_min_ = 1.7°
φ and ω scans	*h* = −35→35
22711 measured reflections	*k* = −14→14
6251 independent reflections	*l* = −26→27

### Refinement

**Table:**

Refinement on *F*^2^	Primary atom site location: structure-invariant direct methods
Least-squares matrix: full	Secondary atom site location: difference Fourier map
*R*[*F*^2^ > 2σ(*F*^2^)] = 0.066	Hydrogen site location: inferred from neighbouring sites
*wR*(*F*^2^) = 0.160	H-atom parameters constrained
*S* = 1.01	*w* = 1/[σ^2^(*F*_o_^2^) + (0.0656*P*)^2^] where *P* = (*F*_o_^2^ + 2*F*_c_^2^)/3
6251 reflections	(Δ/σ)_max_ < 0.001
397 parameters	Δρ_max_ = 0.23 e Å^−^^3^
0 restraints	Δρ_min_ = −0.28 e Å^−^^3^

### Special details

**Table:**

Geometry. All e.s.d.'s (except the e.s.d. in the dihedral angle between two l.s. planes) are estimated using the full covariance matrix. The cell e.s.d.'s are taken into account individually in the estimation of e.s.d.'s in distances, angles and torsion angles; correlations between e.s.d.'s in cell parameters are only used when they are defined by crystal symmetry. An approximate (isotropic) treatment of cell e.s.d.'s is used for estimating e.s.d.'s involving l.s. planes.
Refinement. Refinement of *F*^2^ against ALL reflections. The weighted *R*-factor *wR* and goodness of fit *S* are based on *F*^2^, conventional *R*-factors *R* are based on *F*, with *F* set to zero for negative *F*^2^. The threshold expression of *F*^2^ > σ(*F*^2^) is used only for calculating *R*-factors(gt) *etc*. and is not relevant to the choice of reflections for refinement. *R*-factors based on *F*^2^ are statistically about twice as large as those based on *F*, and *R*- factors based on ALL data will be even larger.

### Fractional atomic coordinates and isotropic or equivalent isotropic displacement parameters (Å^2^)

**Table:**

	*x*	*y*	*z*	*U*_iso_*/*U*_eq_	
C1	0.15141 (12)	0.7594 (3)	0.30121 (18)	0.0559 (9)	
C2	0.16014 (13)	0.6517 (3)	0.28105 (19)	0.0618 (9)	
H2	0.1349	0.5902	0.2705	0.074*	
C3	0.20679 (12)	0.6352 (3)	0.27648 (17)	0.0562 (9)	
H3	0.2130	0.5624	0.2623	0.067*	
C4	0.24445 (11)	0.7262 (3)	0.29288 (15)	0.0425 (7)	
C5	0.23582 (12)	0.8331 (3)	0.31471 (17)	0.0545 (8)	
H5	0.2617	0.8938	0.3269	0.065*	
C6	0.18899 (12)	0.8510 (3)	0.31871 (19)	0.0615 (9)	
H6	0.1828	0.9237	0.3330	0.074*	
C7	0.29051 (12)	0.7746 (3)	0.22679 (17)	0.0502 (8)	
C8	0.33543 (12)	0.7466 (3)	0.21498 (16)	0.0485 (8)	
C9	0.33834 (15)	0.8010 (3)	0.15926 (19)	0.0705 (10)	
H9	0.3132	0.8613	0.1318	0.085*	
C10	0.37794 (16)	0.7665 (4)	0.1447 (2)	0.0827 (12)	
H10	0.3796	0.8031	0.1073	0.099*	
C11	0.41546 (16)	0.6774 (4)	0.1854 (2)	0.0776 (12)	
H11	0.4419	0.6536	0.1746	0.093*	
C12	0.41441 (13)	0.6231 (3)	0.24197 (17)	0.0597 (9)	
H12	0.4401	0.5634	0.2691	0.072*	
C13	0.37438 (11)	0.6583 (3)	0.25844 (16)	0.0460 (7)	
C14	0.33712 (11)	0.6382 (2)	0.32842 (15)	0.0384 (7)	
C15	0.38186 (11)	0.5356 (2)	0.44066 (15)	0.0359 (7)	
C16	0.43022 (11)	0.5918 (2)	0.49417 (16)	0.0434 (7)	
H16	0.4341	0.6728	0.4923	0.052*	
C17	0.47308 (12)	0.5260 (3)	0.55090 (16)	0.0510 (8)	
H17	0.5061	0.5629	0.5876	0.061*	
C18	0.46706 (11)	0.4053 (3)	0.55324 (15)	0.0430 (7)	
H18	0.4958	0.3604	0.5912	0.052*	
C19	0.41816 (11)	0.3536 (2)	0.49879 (15)	0.0346 (6)	
C20	0.37475 (10)	0.4164 (2)	0.44153 (15)	0.0362 (6)	
H20	0.3418	0.3794	0.4048	0.043*	
C21	0.42029 (10)	0.1591 (2)	0.46272 (15)	0.0365 (6)	
C22	0.46375 (11)	0.1061 (2)	0.40343 (15)	0.0402 (7)	
C23	0.49672 (11)	0.1357 (3)	0.37574 (16)	0.0507 (8)	
H23	0.5096	0.2126	0.3803	0.061*	
C24	0.51062 (12)	0.0527 (3)	0.34157 (18)	0.0626 (9)	
H24	0.5332	0.0736	0.3238	0.075*	
C25	0.49123 (13)	−0.0619 (3)	0.33343 (18)	0.0616 (9)	
H25	0.5011	−0.1179	0.3108	0.074*	
C26	0.45743 (11)	−0.0925 (3)	0.35886 (17)	0.0532 (8)	
H26	0.4441	−0.1692	0.3531	0.064*	
C27	0.44305 (10)	−0.0085 (2)	0.39337 (15)	0.0388 (7)	
C28	0.40453 (11)	−0.0402 (3)	0.41594 (16)	0.0432 (7)	
C29	0.35068 (11)	0.0301 (2)	0.46823 (15)	0.0404 (7)	
C30	0.30465 (12)	0.1049 (3)	0.44078 (17)	0.0490 (8)	
H30	0.3016	0.1704	0.4129	0.059*	
C31	0.26329 (13)	0.0809 (3)	0.45527 (19)	0.0628 (9)	
H31	0.2325	0.1315	0.4379	0.075*	
C32	0.26727 (14)	−0.0164 (3)	0.4949 (2)	0.0614 (9)	
C33	0.31256 (14)	−0.0906 (3)	0.52176 (18)	0.0628 (9)	
H33	0.3150	−0.1568	0.5487	0.075*	
C34	0.35451 (12)	−0.0670 (3)	0.50883 (16)	0.0516 (8)	
H34	0.3856	−0.1170	0.5276	0.062*	
Cl1	0.09206 (4)	0.78360 (10)	0.30638 (6)	0.0883 (4)	
Cl2	0.21428 (5)	−0.04566 (12)	0.51094 (7)	0.1140 (5)	
N1	0.29124 (9)	0.7099 (2)	0.28274 (13)	0.0414 (6)	
N2	0.37633 (9)	0.6075 (2)	0.31840 (12)	0.0417 (6)	
N3	0.45216 (9)	0.19030 (19)	0.44073 (12)	0.0405 (6)	
N4	0.39348 (9)	0.05110 (19)	0.45116 (12)	0.0384 (6)	
O1	0.33518 (7)	0.60201 (16)	0.38599 (10)	0.0447 (5)	
O2	0.25236 (9)	0.8446 (2)	0.18965 (12)	0.0715 (7)	
O3	0.40913 (7)	0.23167 (15)	0.50289 (10)	0.0408 (5)	
O4	0.38040 (9)	−0.13455 (18)	0.40336 (13)	0.0649 (7)	

### Atomic displacement parameters (Å^2^)

**Table:**

	*U*^11^	*U*^22^	*U*^33^	*U*^12^	*U*^13^	*U*^23^
C1	0.0412 (16)	0.061 (2)	0.063 (2)	0.0031 (16)	0.0265 (16)	0.0035 (18)
C2	0.0488 (18)	0.049 (2)	0.082 (3)	−0.0122 (16)	0.0319 (18)	−0.0058 (19)
C3	0.0533 (18)	0.0416 (19)	0.068 (2)	−0.0043 (16)	0.0293 (17)	−0.0063 (17)
C4	0.0335 (14)	0.0388 (18)	0.0436 (18)	0.0036 (13)	0.0132 (13)	0.0048 (14)
C5	0.0429 (17)	0.043 (2)	0.069 (2)	−0.0033 (15)	0.0247 (16)	−0.0038 (17)
C6	0.0452 (17)	0.047 (2)	0.082 (3)	0.0029 (16)	0.0282 (17)	−0.0100 (18)
C7	0.0454 (17)	0.048 (2)	0.0401 (19)	−0.0037 (15)	0.0117 (15)	0.0051 (16)
C8	0.0494 (17)	0.051 (2)	0.0359 (18)	−0.0070 (15)	0.0172 (15)	0.0045 (15)
C9	0.069 (2)	0.081 (3)	0.052 (2)	−0.010 (2)	0.0263 (19)	0.013 (2)
C10	0.072 (2)	0.123 (4)	0.054 (2)	−0.017 (3)	0.035 (2)	0.006 (3)
C11	0.075 (2)	0.110 (4)	0.062 (3)	−0.016 (2)	0.046 (2)	−0.013 (2)
C12	0.0563 (19)	0.074 (3)	0.051 (2)	−0.0059 (17)	0.0307 (17)	−0.0092 (18)
C13	0.0439 (16)	0.050 (2)	0.0389 (18)	−0.0101 (14)	0.0189 (14)	−0.0082 (15)
C14	0.0393 (15)	0.0325 (17)	0.0362 (17)	−0.0018 (13)	0.0158 (13)	0.0015 (13)
C15	0.0382 (14)	0.0334 (17)	0.0399 (17)	0.0063 (13)	0.0235 (13)	0.0072 (14)
C16	0.0502 (17)	0.0269 (16)	0.0521 (19)	−0.0039 (13)	0.0270 (15)	−0.0011 (14)
C17	0.0451 (17)	0.043 (2)	0.047 (2)	−0.0094 (14)	0.0135 (15)	−0.0039 (16)
C18	0.0419 (16)	0.0392 (18)	0.0384 (17)	0.0024 (13)	0.0156 (14)	0.0055 (14)
C19	0.0432 (15)	0.0255 (15)	0.0422 (17)	−0.0030 (12)	0.0278 (14)	−0.0001 (13)
C20	0.0329 (14)	0.0360 (17)	0.0365 (16)	−0.0042 (12)	0.0167 (12)	−0.0052 (13)
C21	0.0370 (14)	0.0288 (16)	0.0414 (17)	0.0008 (12)	0.0197 (13)	−0.0009 (13)
C22	0.0365 (14)	0.0397 (18)	0.0420 (17)	−0.0023 (13)	0.0197 (13)	−0.0031 (14)
C23	0.0518 (17)	0.052 (2)	0.054 (2)	−0.0095 (15)	0.0329 (16)	−0.0074 (16)
C24	0.0541 (19)	0.082 (3)	0.063 (2)	−0.0146 (19)	0.0385 (18)	−0.018 (2)
C25	0.0528 (18)	0.069 (2)	0.067 (2)	0.0015 (18)	0.0351 (18)	−0.018 (2)
C26	0.0444 (16)	0.048 (2)	0.064 (2)	−0.0031 (15)	0.0278 (16)	−0.0148 (17)
C27	0.0325 (14)	0.0362 (17)	0.0429 (17)	−0.0004 (12)	0.0171 (13)	−0.0052 (14)
C28	0.0417 (15)	0.0326 (17)	0.0532 (19)	0.0014 (14)	0.0243 (14)	−0.0015 (15)
C29	0.0385 (15)	0.0370 (17)	0.0476 (18)	−0.0055 (13)	0.0247 (14)	−0.0008 (15)
C30	0.0478 (16)	0.0437 (19)	0.060 (2)	0.0019 (15)	0.0320 (16)	0.0043 (16)
C31	0.0513 (19)	0.066 (2)	0.079 (3)	0.0040 (17)	0.0404 (19)	−0.005 (2)
C32	0.065 (2)	0.070 (3)	0.071 (2)	−0.0168 (19)	0.051 (2)	−0.011 (2)
C33	0.073 (2)	0.066 (2)	0.058 (2)	−0.013 (2)	0.0403 (19)	0.0077 (19)
C34	0.0539 (18)	0.046 (2)	0.052 (2)	0.0009 (15)	0.0267 (16)	0.0093 (16)
Cl1	0.0611 (5)	0.0904 (8)	0.1270 (9)	0.0016 (5)	0.0599 (6)	−0.0005 (7)
Cl2	0.1066 (8)	0.1518 (12)	0.1397 (11)	−0.0316 (8)	0.1032 (8)	−0.0167 (9)
N1	0.0382 (12)	0.0372 (14)	0.0401 (14)	0.0015 (11)	0.0156 (11)	0.0056 (12)
N2	0.0447 (13)	0.0413 (15)	0.0408 (14)	0.0015 (11)	0.0243 (12)	0.0008 (12)
N3	0.0452 (13)	0.0337 (14)	0.0461 (15)	−0.0044 (11)	0.0271 (12)	−0.0036 (12)
N4	0.0415 (12)	0.0280 (13)	0.0506 (15)	−0.0043 (10)	0.0280 (12)	−0.0044 (11)
O1	0.0427 (10)	0.0424 (12)	0.0497 (12)	0.0110 (9)	0.0255 (10)	0.0148 (10)
O2	0.0596 (13)	0.0775 (17)	0.0605 (15)	0.0203 (13)	0.0214 (12)	0.0328 (14)
O3	0.0572 (11)	0.0245 (10)	0.0509 (13)	−0.0027 (9)	0.0358 (10)	−0.0013 (9)
O4	0.0691 (14)	0.0408 (13)	0.1020 (19)	−0.0178 (11)	0.0576 (14)	−0.0200 (13)

### Geometric parameters (Å, °)

**Table:**

C1—C2	1.364 (4)	C17—H17	0.9300
C1—C6	1.380 (4)	C18—C19	1.365 (4)
C1—Cl1	1.749 (3)	C18—H18	0.9300
C2—C3	1.377 (4)	C19—C20	1.374 (3)
C2—H2	0.9300	C19—O3	1.419 (3)
C3—C4	1.380 (4)	C20—H20	0.9300
C3—H3	0.9300	C21—N3	1.268 (3)
C4—C5	1.370 (4)	C21—O3	1.349 (3)
C4—N1	1.454 (3)	C21—N4	1.389 (3)
C5—C6	1.377 (4)	C22—C23	1.386 (4)
C5—H5	0.9300	C22—C27	1.394 (4)
C6—H6	0.9300	C22—N3	1.396 (3)
C7—O2	1.221 (3)	C23—C24	1.376 (4)
C7—N1	1.402 (4)	C23—H23	0.9300
C7—C8	1.449 (4)	C24—C25	1.384 (4)
C8—C9	1.392 (4)	C24—H24	0.9300
C8—C13	1.403 (4)	C25—C26	1.372 (4)
C9—C10	1.364 (5)	C25—H25	0.9300
C9—H9	0.9300	C26—C27	1.396 (4)
C10—C11	1.379 (5)	C26—H26	0.9300
C10—H10	0.9300	C27—C28	1.450 (4)
C11—C12	1.380 (5)	C28—O4	1.217 (3)
C11—H11	0.9300	C28—N4	1.415 (3)
C12—C13	1.406 (4)	C29—C34	1.376 (4)
C12—H12	0.9300	C29—C30	1.382 (4)
C13—N2	1.389 (3)	C29—N4	1.453 (3)
C14—N2	1.278 (3)	C30—C31	1.381 (4)
C14—O1	1.336 (3)	C30—H30	0.9300
C14—N1	1.385 (3)	C31—C32	1.364 (5)
C15—C20	1.370 (4)	C31—H31	0.9300
C15—C16	1.372 (4)	C32—C33	1.364 (5)
C15—O1	1.415 (3)	C32—Cl2	1.730 (3)
C16—C17	1.381 (4)	C33—C34	1.374 (4)
C16—H16	0.9300	C33—H33	0.9300
C17—C18	1.385 (4)	C34—H34	0.9300
			
C2—C1—C6	121.4 (3)	C20—C19—O3	117.5 (2)
C2—C1—Cl1	120.1 (3)	C15—C20—C19	117.4 (2)
C6—C1—Cl1	118.5 (3)	C15—C20—H20	121.3
C1—C2—C3	119.0 (3)	C19—C20—H20	121.3
C1—C2—H2	120.5	N3—C21—O3	121.6 (2)
C3—C2—H2	120.5	N3—C21—N4	127.1 (2)
C2—C3—C4	120.4 (3)	O3—C21—N4	111.3 (2)
C2—C3—H3	119.8	C23—C22—C27	118.6 (3)
C4—C3—H3	119.8	C23—C22—N3	119.9 (3)
C5—C4—C3	119.9 (3)	C27—C22—N3	121.5 (2)
C5—C4—N1	120.4 (3)	C24—C23—C22	120.8 (3)
C3—C4—N1	119.6 (3)	C24—C23—H23	119.6
C4—C5—C6	120.3 (3)	C22—C23—H23	119.6
C4—C5—H5	119.9	C23—C24—C25	120.4 (3)
C6—C5—H5	119.9	C23—C24—H24	119.8
C5—C6—C1	119.0 (3)	C25—C24—H24	119.8
C5—C6—H6	120.5	C26—C25—C24	119.8 (3)
C1—C6—H6	120.5	C26—C25—H25	120.1
O2—C7—N1	120.3 (3)	C24—C25—H25	120.1
O2—C7—C8	124.4 (3)	C25—C26—C27	120.0 (3)
N1—C7—C8	115.2 (3)	C25—C26—H26	120.0
C9—C8—C13	120.4 (3)	C27—C26—H26	120.0
C9—C8—C7	121.1 (3)	C22—C27—C26	120.3 (3)
C13—C8—C7	118.5 (3)	C22—C27—C28	120.2 (2)
C10—C9—C8	120.3 (4)	C26—C27—C28	119.4 (3)
C10—C9—H9	119.8	O4—C28—N4	120.5 (3)
C8—C9—H9	119.8	O4—C28—C27	124.7 (3)
C9—C10—C11	120.0 (4)	N4—C28—C27	114.6 (2)
C9—C10—H10	120.0	C34—C29—C30	119.9 (3)
C11—C10—H10	120.0	C34—C29—N4	120.1 (2)
C10—C11—C12	121.1 (3)	C30—C29—N4	119.9 (2)
C10—C11—H11	119.5	C31—C30—C29	119.1 (3)
C12—C11—H11	119.5	C31—C30—H30	120.5
C11—C12—C13	119.8 (3)	C29—C30—H30	120.5
C11—C12—H12	120.1	C32—C31—C30	120.5 (3)
C13—C12—H12	120.1	C32—C31—H31	119.8
N2—C13—C8	122.8 (3)	C30—C31—H31	119.8
N2—C13—C12	118.8 (3)	C31—C32—C33	120.5 (3)
C8—C13—C12	118.3 (3)	C31—C32—Cl2	119.5 (3)
N2—C14—O1	123.0 (2)	C33—C32—Cl2	120.0 (3)
N2—C14—N1	126.4 (3)	C32—C33—C34	119.8 (3)
O1—C14—N1	110.6 (2)	C32—C33—H33	120.1
C20—C15—C16	122.2 (3)	C34—C33—H33	120.1
C20—C15—O1	117.5 (2)	C33—C34—C29	120.3 (3)
C16—C15—O1	120.1 (2)	C33—C34—H34	119.9
C15—C16—C17	118.9 (3)	C29—C34—H34	119.9
C15—C16—H16	120.5	C14—N1—C7	119.7 (2)
C17—C16—H16	120.5	C14—N1—C4	122.3 (2)
C16—C17—C18	120.2 (3)	C7—N1—C4	117.9 (2)
C16—C17—H17	119.9	C14—N2—C13	116.1 (2)
C18—C17—H17	119.9	C21—N3—C22	117.0 (2)
C19—C18—C17	118.7 (3)	C21—N4—C28	119.3 (2)
C19—C18—H18	120.6	C21—N4—C29	122.7 (2)
C17—C18—H18	120.6	C28—N4—C29	117.8 (2)
C18—C19—C20	122.6 (2)	C14—O1—C15	116.9 (2)
C18—C19—O3	119.8 (2)	C21—O3—C19	116.43 (19)
			
C6—C1—C2—C3	−1.5 (5)	C34—C29—C30—C31	0.5 (4)
Cl1—C1—C2—C3	179.2 (3)	N4—C29—C30—C31	177.5 (3)
C1—C2—C3—C4	0.7 (5)	C29—C30—C31—C32	−1.3 (5)
C2—C3—C4—C5	0.8 (5)	C30—C31—C32—C33	1.0 (5)
C2—C3—C4—N1	−175.6 (3)	C30—C31—C32—Cl2	−178.9 (2)
C3—C4—C5—C6	−1.5 (5)	C31—C32—C33—C34	0.0 (5)
N1—C4—C5—C6	174.9 (3)	Cl2—C32—C33—C34	180.0 (3)
C4—C5—C6—C1	0.7 (5)	C32—C33—C34—C29	−0.8 (5)
C2—C1—C6—C5	0.9 (5)	C30—C29—C34—C33	0.5 (4)
Cl1—C1—C6—C5	−179.9 (2)	N4—C29—C34—C33	−176.5 (3)
O2—C7—C8—C9	1.0 (5)	N2—C14—N1—C7	−12.6 (4)
N1—C7—C8—C9	177.5 (3)	O1—C14—N1—C7	168.3 (2)
O2—C7—C8—C13	−175.6 (3)	N2—C14—N1—C4	170.8 (3)
N1—C7—C8—C13	1.0 (4)	O1—C14—N1—C4	−8.3 (3)
C13—C8—C9—C10	2.1 (5)	O2—C7—N1—C14	−174.7 (3)
C7—C8—C9—C10	−174.3 (3)	C8—C7—N1—C14	8.7 (4)
C8—C9—C10—C11	−0.2 (6)	O2—C7—N1—C4	2.1 (4)
C9—C10—C11—C12	−1.0 (6)	C8—C7—N1—C4	−174.6 (2)
C10—C11—C12—C13	0.3 (5)	C5—C4—N1—C14	112.2 (3)
C9—C8—C13—N2	174.8 (3)	C3—C4—N1—C14	−71.4 (3)
C7—C8—C13—N2	−8.7 (4)	C5—C4—N1—C7	−64.5 (4)
C9—C8—C13—C12	−2.8 (4)	C3—C4—N1—C7	112.0 (3)
C7—C8—C13—C12	173.8 (3)	O1—C14—N2—C13	−176.1 (2)
C11—C12—C13—N2	−176.1 (3)	N1—C14—N2—C13	4.8 (4)
C11—C12—C13—C8	1.6 (5)	C8—C13—N2—C14	6.0 (4)
C20—C15—C16—C17	−0.2 (4)	C12—C13—N2—C14	−176.5 (3)
O1—C15—C16—C17	173.9 (2)	O3—C21—N3—C22	176.2 (2)
C15—C16—C17—C18	0.1 (4)	N4—C21—N3—C22	−2.9 (4)
C16—C17—C18—C19	−0.2 (4)	C23—C22—N3—C21	177.8 (3)
C17—C18—C19—C20	0.3 (4)	C27—C22—N3—C21	−2.5 (4)
C17—C18—C19—O3	−175.2 (2)	N3—C21—N4—C28	5.0 (4)
C16—C15—C20—C19	0.3 (4)	O3—C21—N4—C28	−174.2 (2)
O1—C15—C20—C19	−174.0 (2)	N3—C21—N4—C29	−169.8 (3)
C18—C19—C20—C15	−0.3 (4)	O3—C21—N4—C29	11.0 (3)
O3—C19—C20—C15	175.3 (2)	O4—C28—N4—C21	−178.0 (3)
C27—C22—C23—C24	−2.5 (4)	C27—C28—N4—C21	−1.5 (4)
N3—C22—C23—C24	177.2 (3)	O4—C28—N4—C29	−2.9 (4)
C22—C23—C24—C25	0.8 (5)	C27—C28—N4—C29	173.5 (2)
C23—C24—C25—C26	0.7 (5)	C34—C29—N4—C21	−130.7 (3)
C24—C25—C26—C27	−0.5 (5)	C30—C29—N4—C21	52.3 (4)
C23—C22—C27—C26	2.7 (4)	C34—C29—N4—C28	54.4 (4)
N3—C22—C27—C26	−177.0 (3)	C30—C29—N4—C28	−122.6 (3)
C23—C22—C27—C28	−174.8 (3)	N2—C14—O1—C15	5.8 (4)
N3—C22—C27—C28	5.5 (4)	N1—C14—O1—C15	−175.0 (2)
C25—C26—C27—C22	−1.2 (4)	C20—C15—O1—C14	−103.7 (3)
C25—C26—C27—C28	176.3 (3)	C16—C15—O1—C14	82.0 (3)
C22—C27—C28—O4	173.0 (3)	N3—C21—O3—C19	20.3 (4)
C26—C27—C28—O4	−4.5 (4)	N4—C21—O3—C19	−160.5 (2)
C22—C27—C28—N4	−3.3 (4)	C18—C19—O3—C21	−98.9 (3)
C26—C27—C28—N4	179.3 (2)	C20—C19—O3—C21	85.4 (3)

### Hydrogen-bond geometry (Å, °)

**Table:**

*D*—H···*A*	*D*—H	H···*A*	*D*···*A*	*D*—H···*A*
C20—H20···O2^i^	0.93	2.34	3.234 (3)	162

Symmetry codes: (i) −*x*+1/2, *y*−1/2, −*z*+1/2.
